# Fan-out routing and optical splitting techniques for compact optical interconnects using single-mode polymer waveguides

**DOI:** 10.1080/09500340.2014.983197

**Published:** 2014-11-25

**Authors:** Kevin L. Kruse, Christopher T. Middlebrook

**Affiliations:** ^a^ Electrical and Computer Engineering Department, Michigan Technological University, Houghton, MI, USA

**Keywords:** optical interconnects, polymer waveguides, waveguide devices

## Abstract

Polymer waveguide (WG) S-bends are necessary for fan-out routing techniques and optical splitting in high-density optical interconnects. Designing and manufacturing of optimal S-bends are critical for minimizing optical link loss while maintaining overall size and layout constraints. Complete structural loss analysis is demonstrated theoretically and shown experimentally utilizing both radial and transitional loss in single-mode (SM) polymer WG radial arc, cosine, and raised-sine S-bend profiles. SM polymer WG straights were first fabricated to measure standard propagation loss. SM WG S-bends were fabricated incorporating straight lead-in and lead-out sections to incorporate transitional loss present in workable designs. S-bend designs were measured at different dimensions and matched to theoretical losses. Compact cosine and radial arc S-bends exhibited the lowest structure loss for low and high NA WGs, respectively. High-speed performance of SM WG straights and S-bends was measured at 10 Gbit/s demonstrating low error rate. Optical splitters designed with S-bends and tapers were also evaluated and fabricated. Trade-off between optimal loss and minimal device size is discussed.

## 1. Introduction

Single-mode (SM) polymer waveguides (WGs) are critical for realizing high-speed optical interconnects, allowing for data transmission rates greater than 10 Gbits/s. Polymer WGs do not exhibit the high levels of cross-talk and transmission loss present in high-density copper interconnects at frequencies beyond 1 GHz [1]. While SM polymer WGs are designed to exhibit low losses, they are also geometrically constrained to the application’s required footprint. S-bends are curved WG designs that efficiently optimize both structural losses and bending lengths [2].

**Figure 1.**
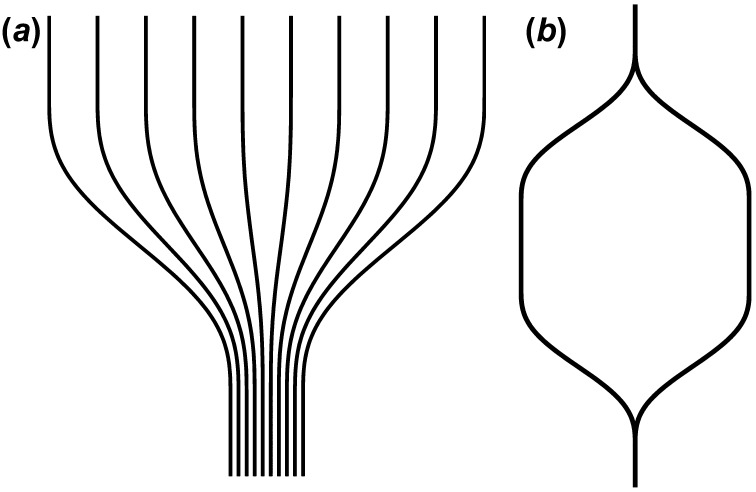
Images of S-bends utilized in WG fan-out (*a*) and MZI (*b*) designs.

S-bends are also optimal for many specific WG-integrated devices in addition to routing WGs around structures. S-bends can be used to adjust WG density within an optical data bus and in the design of optical splitters and couplers, an important component in integrated Mach-Zehnder Interferometers (MZIs). The possible applications for S-Bends include optical interconnects [3,4], optical signal modulation [4], environmental sensing [5,6], and silicon-organic hybrid interconnects [7]. Practical S-bend designs for these applications are shown in Figure 1.

Previously published journal articles proposed solutions for minimizing optical loss while obtaining high optical density with SM WG S-bend structures. The utilization of polyline-based S-bends [8] and radial tapers [9] to optimize WG designs has been proposed. Polyline-based S-bends and radial tapers are complex and difficult structures to design and scale for integration into photolithographic masks. Other publications [10–12] have shown that sine-derived S-bends of equivalent dimensions offer similar levels of loss in simple, flexible, and expandable formulas. Previous works mentioned are purely theoretical without any experimental results to verify and compare different S-bend designs.

WG S-bends have been previously fabricated using a variety of materials. Semiconductor-based devices have been fabricated with MBE-grown epilayering [13] and etching [3] techniques. Titanium-doped Lithium Niobate S-bends were fabricated, tested, and correlated to theoretical losses [2,14]. Experimental results for polymer-based SM WG S-bends have been reported using the radial arc design with an emphasis on cross-talk measurements [15]. Prior journals assume only one type of S-bend design for their builds and do not discuss optimal S-bend profiles required for high degrees of integration density.

This paper experimentally compares the structure loss of fabricated S-bend designs. Results are correlated with acknowledged structure loss theory for the design of compact S-bends and splitters. Step-index SM WG S-bends were fabricated with various dimensions and correlated to theoretical loss. The bit error rate testing of fabricated SM straights and S-bend structures at 10 Gbit/s was conducted. Optical splitters designed using SM WG S-bends and adiabatic tapers were also fabricated and evaluated to determine optimal splitting efficiency. Finally, a brief discussion of the trade-off between optimal loss and minimal package footprint requirements for both S-bends and splitters follows.

## 2. S-Bend theory

**Figure 2.**
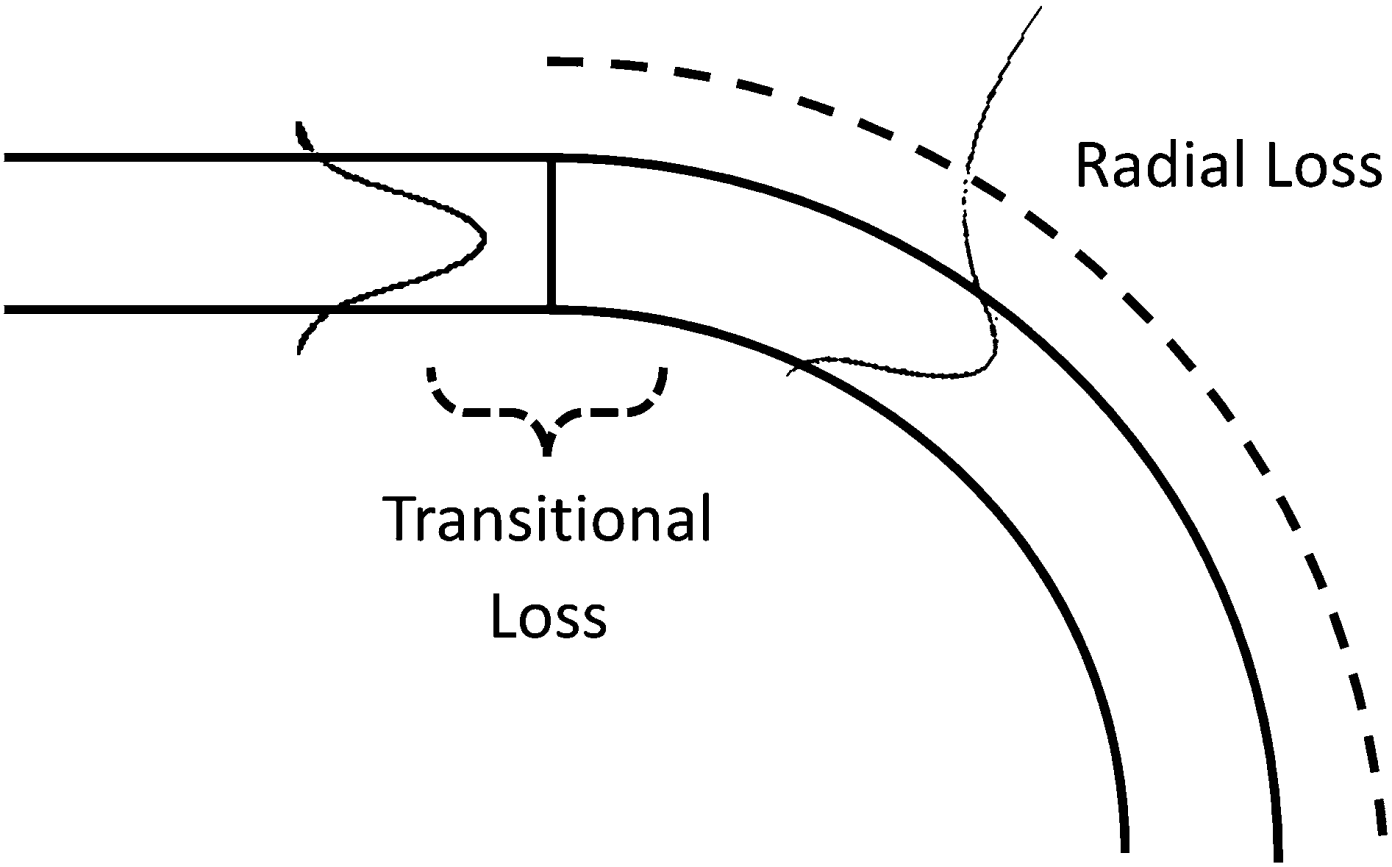
Transitional and radial loss regions of a propagating mode in a WG bend.

S-bend structure loss can be decomposed into length-dependent radial loss and junction-localized transitional loss, as illustrated in Figure 2. Radial loss [16–18] is the result of continuous power radiation along the entire WG bend due to the asymmetric shift of the guided mode into the caustic region. Transitional loss [19,20] is the result of coupling loss that arises from modal mismatch at straight/bend interfaces. Both loss mechanisms have been thoroughly studied in the previous literature and established as complex functions of the WG’s physical properties.

Low numerical aperture (NA), SM polymer WGs experience low levels of modal confinement, strongly impacting minimal device size for low-loss SM polymer WG builds. Thus, the chosen NA impacts the overall link loss of SM WG S-bend designs. Previous findings on radial and transitional loss are briefly summarized and applied toward S-bend structure loss analysis.

### 2.1. Radial loss

Radial loss is a result of the limited guidance of the propagating mode due to speed limitations beyond the outer end of the WG bend known as the caustic region. The radial attenuation coefficient, *α*(*R,s*), a guided mode exhibits per unit length *s* in a bend radius *R* is expressed by Equation (1). *C*_1_ and *C*_2_ are radially independent loss coefficients that are dependent on the WG’s NA and size [14,21].

(1) $$\alpha \left(R,s\right)=C_{1}\exp\;\left(-C_{2}R\left(s\right)\right)$$

The radius of curvature function, *R*(*s*), of the S-bend profile, *y*(*x*), is required to determine the total radial loss and is obtained using Equation (2). By implementing Equation (2) into Equation (1), the normalized radial loss, Γ_*R*_, is determined by the integration of *α*(*R*(*s’*)*, s’*) over the length of the S-bend. The radial loss function is shown in Equation (3), where *s’* and *S* are the incremental and total length of the S-bend [3].

(2) $$R\left(s\right)=\frac{{\left(1+y^{\prime }{\left(x\right)}^{2}\right)}^{\frac{3}{2}}}{\left|y^{\prime \prime }\left(x\right)\right|}$$

(3) $$\Gamma_{R}=\exp\left(-\int_{S}^{0}\alpha \left(R\left(s^{\prime }\right),s^{\prime }\right)ds^{\prime }\right)$$

### 2.2. Transitional loss

Transitional loss arises when there are discontinuities within the WG’s curvature, where abrupt shifts in the propagation modal profile occur. Guided modes within curved WGs, in comparison to straight WGs, are wider and shifted toward the outside of the curve, as shown in Figure 2. At the intersection of a straight and radial bend, modal mismatch loss occurs as the guided mode transitions to its new steady state. The normalized transitional loss, Γ_*T*_, is determined by the overlap integral [21] between the input and output electric fields, *E*_in_ and *E*_out_, as shown in Equation (4).

(4) $$\Gamma_{T}=1-\frac{{\left|\;\int \int \;E_{\text{in}}\left(x,y\right)\;E_{\text{out}}^{\text{*}}\left(x,y\right)dydx\right|}^{2}}{\;\int \int \;{\left|E_{\text{in}}\left(x,y\right)\right|}^{2}dxdy\;\int \int \;{\left|E_{\text{out}}\left(x,y\right)\right|}^{2}dxdy}$$

As the WG’s radius of curvature decreases, the radial mode widens and linearly shifts away from the WG’s center as a function of wavelength *λ*, WG width *w*, and effective refractive index *n*_eff_. Taking into account this modal shift in WG bends for symmetric WGs, transitional loss has been analyzed and simplified by previous authors [19,20]. These studies note an inverse quadratic relationship between bend radius and the resulting transitional loss in decibels at the straight-bend interface, as shown in Equation (5).

(5) $$\Gamma_{T,dB}=T_{1}R^{-2},\;T_{1}\propto {\left(\frac{\pi^{2}n_{\text{eff}}^{2}w^{3}}{\lambda^{2}}\right)}^{2}$$

### 2.3. Total structure loss

Bend structure designs can have multiple regions of transitional and radial loss. Radial loss occurs wherever the radius of curvature is not infinite. Transitional losses are incorporated where there are discontinuities in the S-bend’s curvature profile. Assuming the individual normalized outputs are independent from one another, the product of all the normalized outputs results in the total structure efficiency. Converting the loss into terms of dB the total normalized structure loss is the summation of the normalized loss elements, as shown in Equation (6).

(6) $$\text{Total Loss}\left(\text{dB}\right)=10\log_{10}\left(\prod \Gamma_{R}\prod \Gamma_{T}\right)=\sum \Gamma_{R,dB}+\sum \Gamma_{T,dB}$$

## 3. S-Bend design

**Figure 3.**
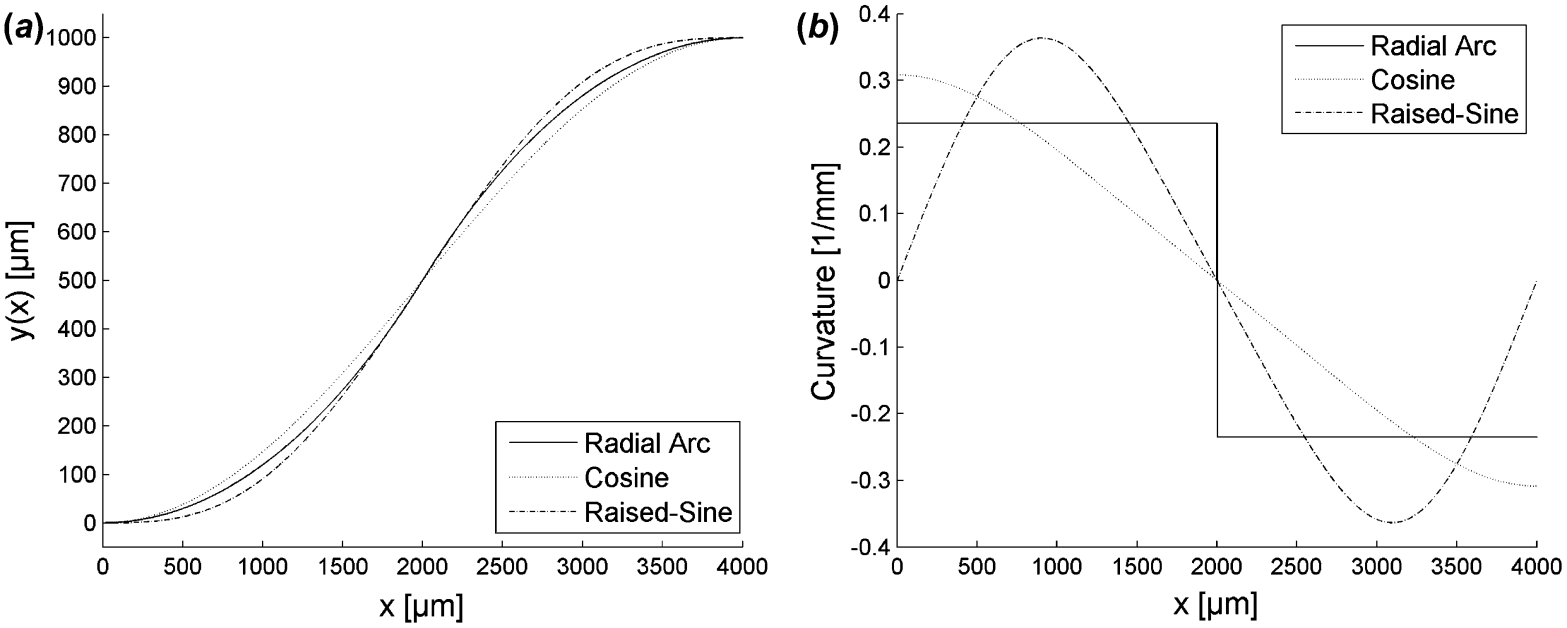
S-Bend profiles (*a*) and their corresponding curvature functions (*b*).

Sine-derived S-bends are designed from one of three profiles: radial arc, cosine, and raised-sine S-bends. These profiles can easily be expanded to a designated width, *W*, and length, *L*. Radial arc S-bends are formed using two inverted arcs of constant curvature, calculated by Equation (7), that intersect at the arcs’ tangents. Cosine and raised-sine S-bends are formed using the sinusoidal functions stated in Equations (8) and (9). These profile’s functions and their associated curvature functions, the inverse of *R*(*s*), are both expressed in Figure 3.

(7) $$y\left(x\right)=\left\{\begin{array}{cc} \sqrt{R^{2}-x^{2}}-\left(R-W\right), & \text{where}\;0\leq x\leq L/2\\ -\sqrt{R^{2}-{\left(x-L\right)}^{2}}+R, & \text{where}\;L/2\leq x\leq L \end{array}\right.,\;R=\frac{L^{2}}{4W}\left(1+\frac{W^{2}}{L^{2}}\right)$$

(8) $$y\left(x\right)=\frac{W}{2}\left(1-\cos\left(\frac{\pi }{L}x\right)\right),\;\text{where}\;0\leq x\leq L$$

(9) $$y\left(x\right)=\frac{W}{L}x-\frac{W}{2\pi }\sin\left(\frac{2\pi }{L}x\right),\;\text{where}\;0\leq x\leq L$$

According to the theoretical work of Mustieles [12] and Kumar [11], cosine S-bends have the lowest loss followed by radial arc and then raised-sine S-bends. However, their work is based purely off of radial loss alone and fails to integrate the transitional losses present at the S-bend interfaces. As most S-bends are directly connected to WG straights in practical applications, transitional loss plays a large role in the S-bend’s total structure loss. A complete loss analysis of the overall S-bend structure including the impact from both radial and transitional loss is needed.

SM polymer WGs are manufactured with a low refractive index (RI) contrast to minimize coupling loss with optical fibers. The low RI contrast decreases the amount of modal confinement and increases the amount of the propagating mode shifts outward within WG bends. Radial arc and cosine S-bends both suffer heightened levels of transitional loss at their end faces. Radial arc S-bends also suffer transitional loss at the intersection between its inverted radial arcs. Raised-sine S-Bends, in contrast, experience no modal mismatch due to its smooth curvature profile and zero curvature at the end faces, *y*(*0*) and *y*(*L*) [14].

Total structure loss of SM polymer WG S-bends is evaluated using theoretical equations and rigorous simulations to determine optimal S-bend configurations and dimensions. The Beam Propagation Method (BPM) was used to simulate the radial and transitional losses, both as a function of radius of curvature, for symmetric WGs of various NA and size that fulfill SM functionality. These values were then utilized to solve for the total structure loss for the three S-bend designs: radial arc, cosine, and raised-sine.

**Figure 4.**
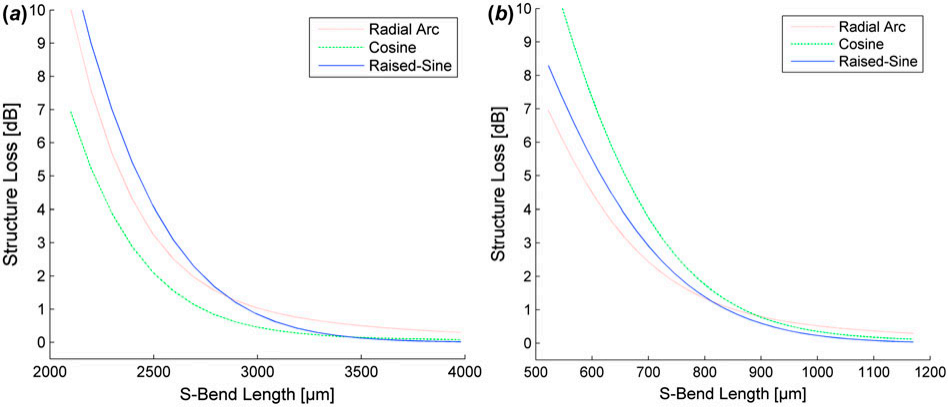
Simulated loss for SM WGs S-Bends (*W* = 0.5 mm) of NA = 0.15 (*a*) and NA = 0.35 (*b*). (The colour version of this figure is included in the online version of the journal.)

**Figure 5.**
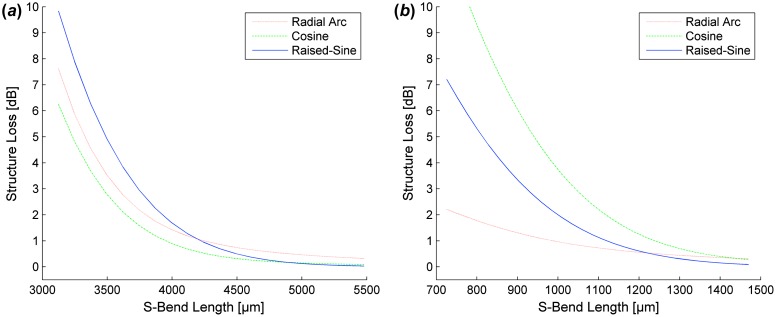
Simulated loss for SM WGs S-Bends (*W* = 1.0 mm) of NA = 0.15 (*a*) and NA = 0.35 (*b*). (The colour version of this figure is included in the online version of the journal.)

S-bend structure loss was simulated for SM WGs of varying NA (0.15–0.35) to show S-bend efficiency for various SM WG characteristics. To maintain SM functionality, the dimensions of the SM WGs of various NAs were calculated using the effective index method [23]. Simulated losses for S-bend structures of various widths, lengths, and NAs are shown in Figures 4 and 5.

Raised-sine S-bends exhibit the shortest length requirements to obtain minimal (<0.1 dB) structure loss regardless of the WG’s NA. However, there is negligible difference in structure loss when comparing S-bend designs in this range. S-bend design selection is more important when footprint requirements are smaller than allowed for minimal structure loss. Reducing length requirements, the most efficient S-bend designs are cosine and radial arc S-bends for low NA and high NA WGs, respectively.

## 4. Fabrication and evaluation

In this work, UV-Cured Optical Elastomers received from Dow Corning® were utilized for SM WG fabrication. The polymers exhibit both low loss at *λ* = 850 (< 0.04 dB/cm) and *λ* = 1310 (< 0.4 dB/cm) [24] and minimal birefringence. They are also capable of resisting harsh environments, including high-temperatures and high-humidity tests [24,25], are compatible with PCB processes [26,27], and can be easily integrated into large-scale manufacturing [28]. These qualities ensure that this WG polymer is capable of being successfully embedded into PCBs as cost-effective chip-to-chip and board-to-board optical interconnects with minimal device degradation or failure.

SM polymer WGs (6 μm × 6 μm) were desired for high-speed optical interconnects and efficient SMF connectivity. Isotropic SM WGs with symmetric cross-sections do not exhibit polarization-dependent losses [29]. The material’s NA was tailored at 0.15 to exhibit SM functionality at the desired dimensions. This low NA allows for the fabrication of 6-μm SM WGs which exhibit minimal coupling loss and high alignment tolerances for connectorization with SMFs and VCSELs.

**Figure 6.**
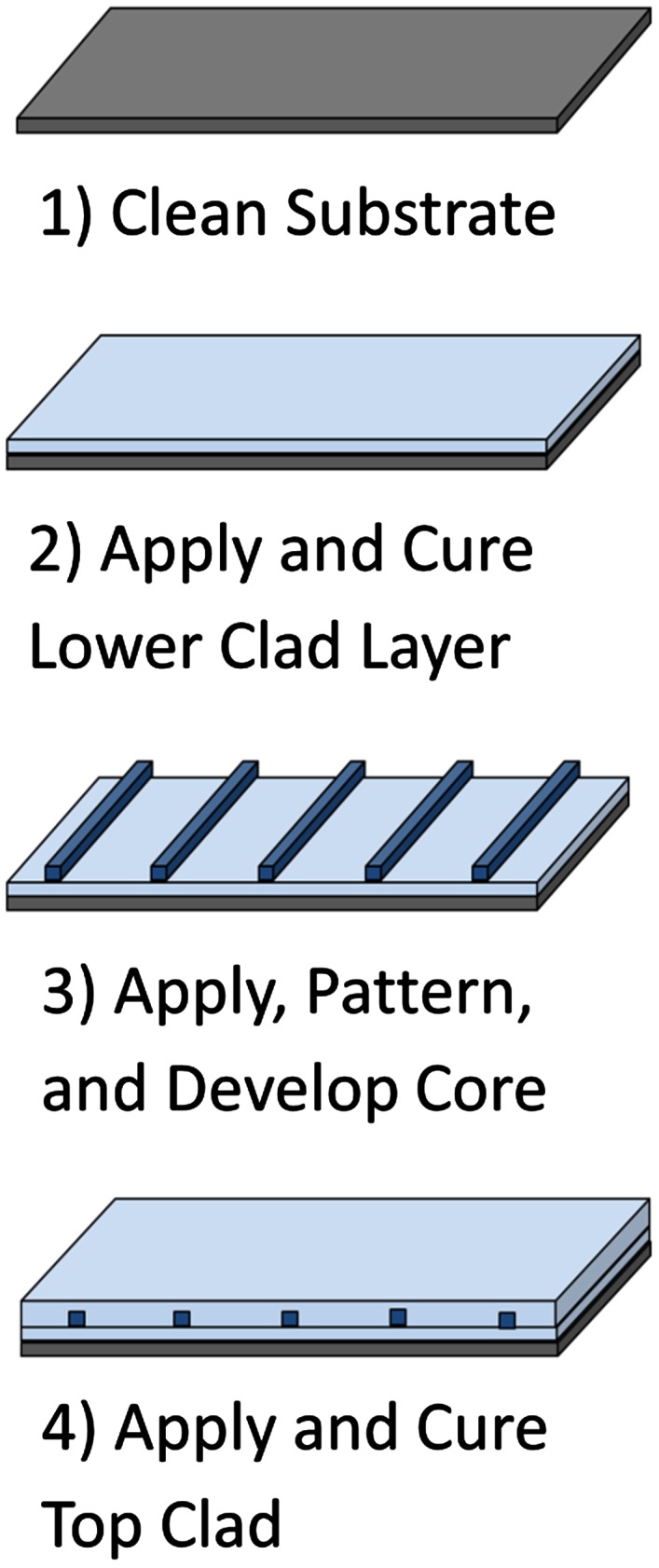
Fabrication procedure for SM polymer WGs. (The colour version of this figure is included in the online version of the journal.)

SM WGs are fabricated using traditional photolithographic processes using silicone materials from Dow Corning^®^. Using a cleaned FR-4 substrate, a layer of low RI cladding polymer is spin-coated, pre-baked, UV-cured, and post-baked to drive the polymerization to completion. A layer of higher RI core material is spin-coated to the appropriate thickness of 6 μm and pre-baked. WGs 6 μm in width were UV-exposed using a high-resolution chrome-on-quartz mask and post-baked. The non-polymerized material was washed away using an appropriate solvent. Finally, a top clad was applied and polymerized accordingly. The polymer SM WG fabrication procedure is shown in Figure 6.

Six micrometer WG straights were fabricated to show material functionality for SM applications. WG facets were cleaved and polished using a PCB milling platform, and index-matching fluid was applied during testing to minimize coupling loss. Optical testing was done through the use of the end-fire coupling method with SMFs (MFD = 9.2 μm) at *λ* = 1310 nm. The cut-back method was conducted on SM WG straights to obtain WG propagation loss due to material absorption and scattering [30].

**Figure 7.**
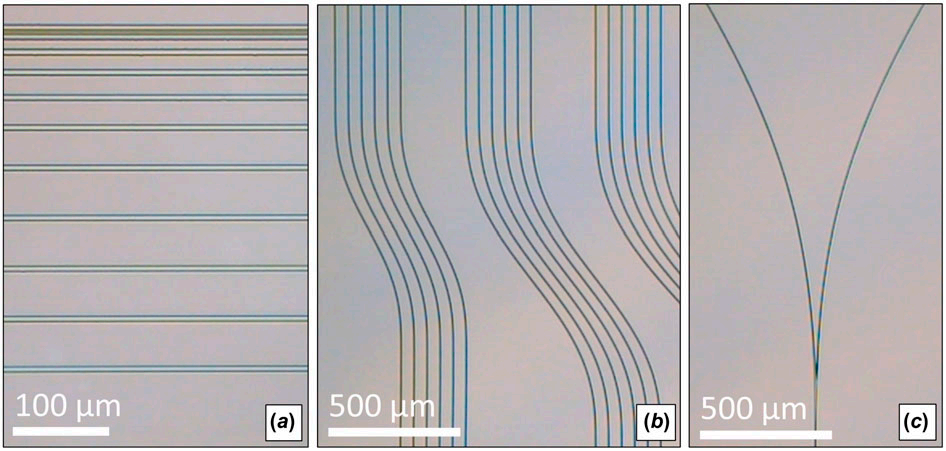
Top–down view of 6-μm SM WG straights (*a*), raised-sine S-bends (*b*), and splitters (c). (The colour version of this figure is included in the online version of the journal.)

Radial arc, cosine, and raised-sine S-bends were fabricated at various widths (500–1000 μm) and lengths (2000–6000 μm). Optical splitters were manufactured using S-bends and tapers. Fabricated WG straights, S-bends, and splitters are shown in Figure 7. The structure losses for S-bends and splitters were evaluated after omitting known propagation loss.

## 5. Results and discussion

### 5.1. SM WG straights

WG straights physically measured with the end-fire coupling method incur both WG propagation and SMF coupling loss. Since coupling loss is independent of WG length, propagation loss can be isolated by plotting optical loss as a function of WG length. Conducting the cut-back method for 16 fabricated SM WGs, propagation loss of 0.59 ± 0.02 dB/cm was measured.

**Figure 8.**
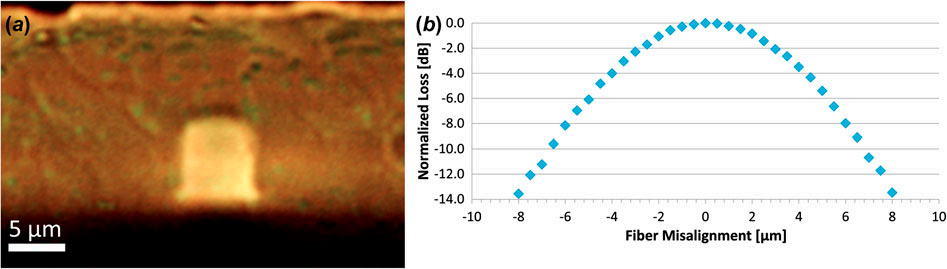
Cross-section (*a*) and misalignment loss (*b*) for a 6 × 6 μm SM WGs. (The colour version of this figure is included in the online version of the journal.)

Step-index polymer SM WGs exhibit low coupling loss when fabricated at a low NA similar to those of SMFs. Measured using the cut-back method, coupling loss per WG/SMF junction was 0.06 dB. This high degree of coupling is paired with high misalignment tolerances. The output power of 6-μm SM WGs was measured in relation to SMF displacement, as shown in Figure 8. The SM WG’s 1 dB and 3 dB alignment tolerances were ±2.0 μm and ±3.5 μm, respectively.

### 5.2. SM WG S-Bends

**Figure 9.**
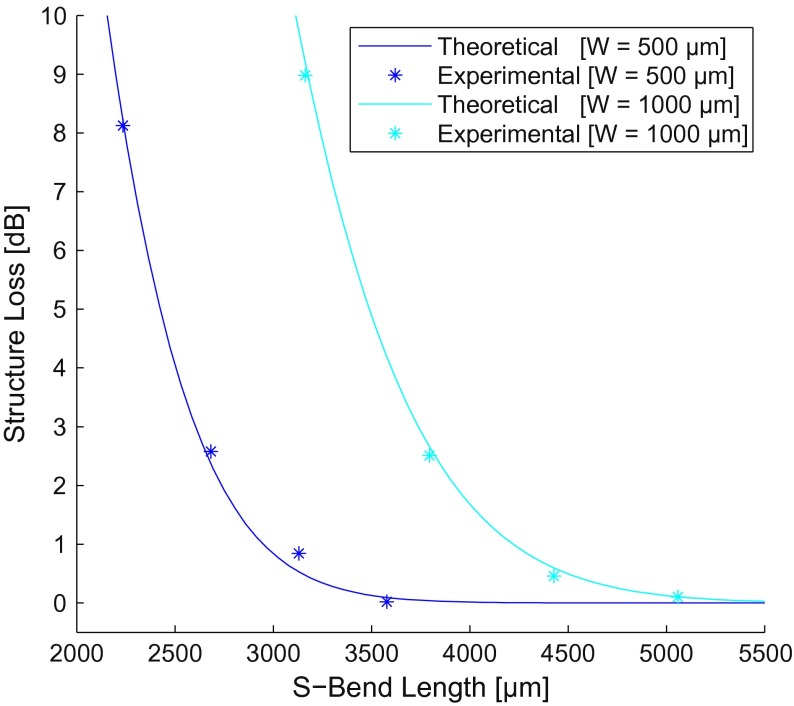
Raised-sine structure loss for theoretical (lines) and experimental (stars; *n* = 6) S-bends. (The colour version of this figure is included in the online version of the journal.)

Raised-sine S-bends theoretically exhibit no transitional loss, shown previously in Figure 3, and can be utilized to find the radial loss coefficients for fabricated SM polymer WGs. Raised-sine S-bend radial loss was isolated from experimental losses after omitting WG propagation and SMF coupling loss. Theoretical loss curves showed strong correlation when fit to experimental results, as shown in Figure 9. Fitted theoretical curves resulted in radially independent loss coefficients of *C*_1_ = 0.053 (*unitless*) and *C*_2_ = 2.3 mm^−1^.

**Figure 10.**
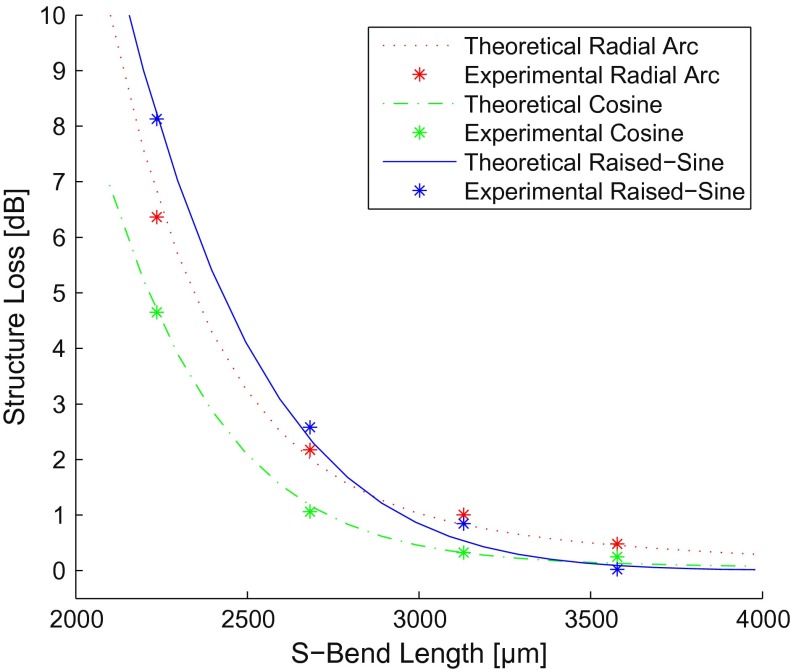
S-bend structure loss for theoretical (lines) and experimental (stars; *n* = 6) results. (The colour version of this figure is included in the online version of the journal.)

Knowing the radial loss constants, the transitional loss coefficient for S-bend structures can now be isolated. Radial arc, cosine, and raised-sine S-bends were fabricated and tested to compare structure loss, shown in Figure 10. Fitted theoretical curves for radial arc and cosine S-bends gave us a transitional loss coefficient of *T*_1_ = 1.6 mm^2^.

The structure losses for all three devices are quite comparable while still exhibiting loss characteristics based on the ratio of radial-to-transitional loss present in each structure. Raised-sine S-bend structure loss is solely dependent on exponential-based radial loss. While the raised-sine profile is the least efficient design at shorter S-bend lengths, its structure loss curve approaches minimal loss at the fastest rate as the S-bend length increases. In contrast, the radial arc design heavily relies on the quadratically dependent transitional loss and exhibits higher levels of structure loss at longer S-bend lengths. Cosine S-bends exhibit the lowest loss at shorter S-bend lengths since this design balances radial and transitional loss, both which increase nonlinearly with decreasing bend radius.

**Figure 11.**
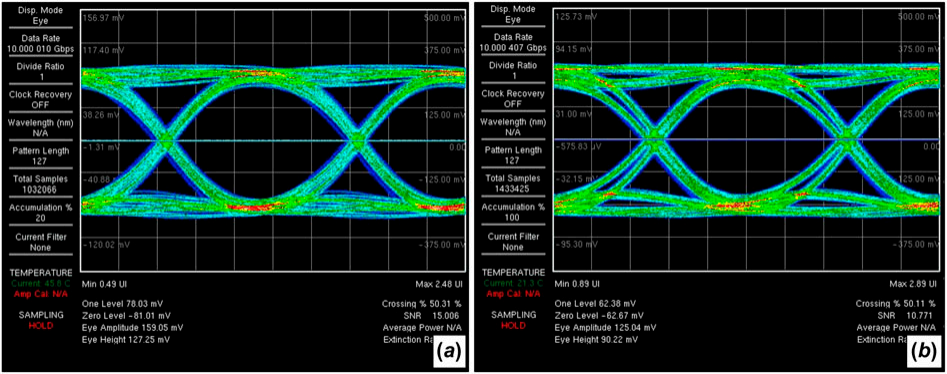
Eye diagrams at 10 Gbits/s for SM WGs (*a*) and SM WGs with S-bends (*b*). (The colour version of this figure is included in the online version of the journal.)

Effective data transmission with SM WG designs is important for their realization as high-bandwidth optical interconnects. Bit error rate testing at 10 Gbits/s was conducted on 3-cm-long samples with both SM WG straights and raised-sine S-bends. As shown in Figure 11, both devices show open-eye diagrams and effective high-speed data transmission.

### 5.3. SM WG optical splitters

Optical splitters utilize S-bends for compact designs for both power distribution and MZIs. While an optical splitter can be manufactured in the form of a “Y-branch,” the small-angle requirement (<2°) for efficient light division requires a long splitter length to obtain minimal splitting loss [31]. Utilizing S-bends for optical splitting significantly reduces the required footprint of the splitter.

Optical splitters utilizing shortened, 1 dB and 3 dB loss raised-sine S-bends were fabricated and measured. Optical testing of photolithographic splitters showed balanced levels of optical power splitting with a measured spitting ratio of 49:51 ± 2%. The shortened WG-to-S-bend splitters exhibited >3 dB of splitting loss, loss not accounted for propagation loss or S-bend structure loss. Splitting loss is due to S-bend overlapping and WG widening before optical splitting occurs. Not only does this cause a discontinuity in the radius of curvature, introducing transitional loss, but it also starts the S-bend at an offset angle. Longer S-bends would result in lower levels of overlap discontinuity and lower splitting loss.

**Figure 12.**
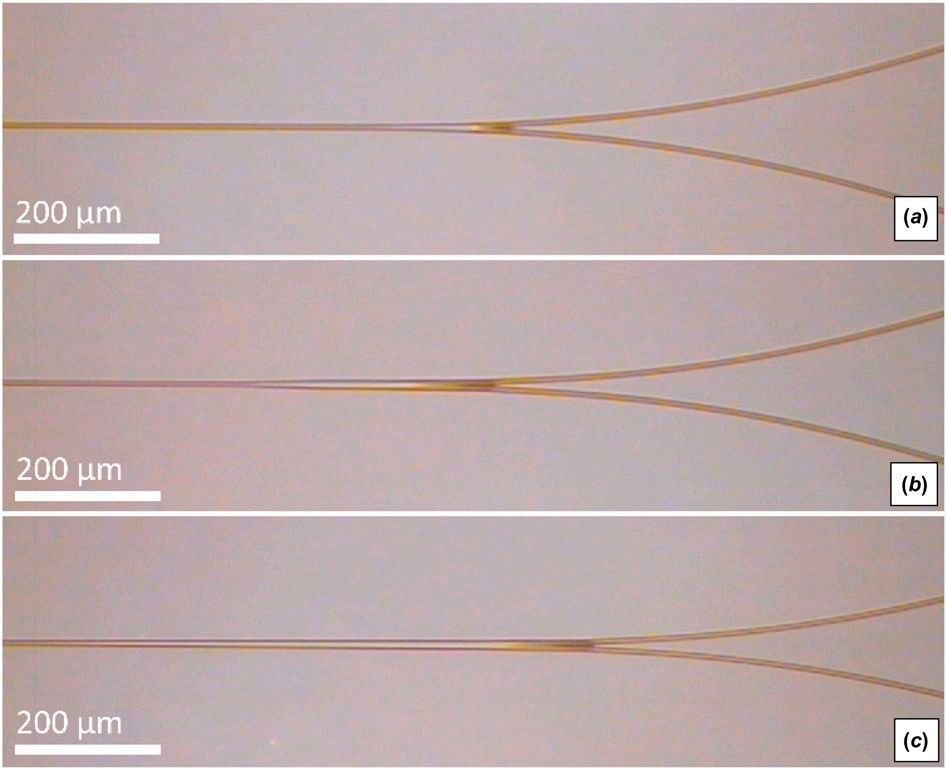
Topdown view of WG S-bend splitters integrated with no splitting taper (*a*), a 100-μm-long taper (*b*), and a 500-μm-long taper (*c*). (The colour version of this figure is included in the online version of the journal.)

**Figure 13.**
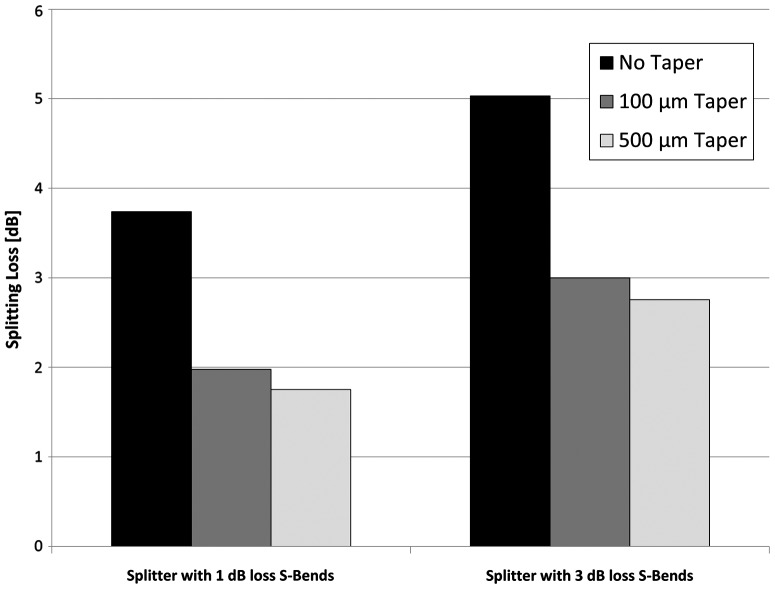
Splitting loss as a function of S-bend type and taper length.

Adiabatic tapers can be utilized as an alternative method to improve splitting efficiencies while minimizing device size. Splitting tapers 100 μm and 500 μm long were inserted between the straight/S-bend interfaces, as shown in Figure 12. The taper widens the fundamental mode before coupling with S-bend splitters. This modal expansion removes unwanted S-bend overlap, reduces transitional loss, and minimizes overall splitting loss. The experimental splitting losses for the taper designs are shown in Figure 13. The inclusion of 100-μm and 500-μm-long tapers in S-bend splitters reduced splitting loss by 1.9 dB and 2.1 dB, respectively.

### 5.4. Footprint requirements

The structure loss of SM WG S-bends of constant width is strongly dependent on device length. For S-bends of various widths, equivalent structure loss is obtained when a quadratic ratio between S-bend length and width is maintained, as described by Equation (10). This demonstrates declining length requirements for equivalent loss when S-bend structures with larger widths are required. This relationship in S-bend builds has been observed by previous authors [14,32].

(10) $$\text{Loss}\propto \frac{L}{\sqrt{W}}$$

**Figure 14.**
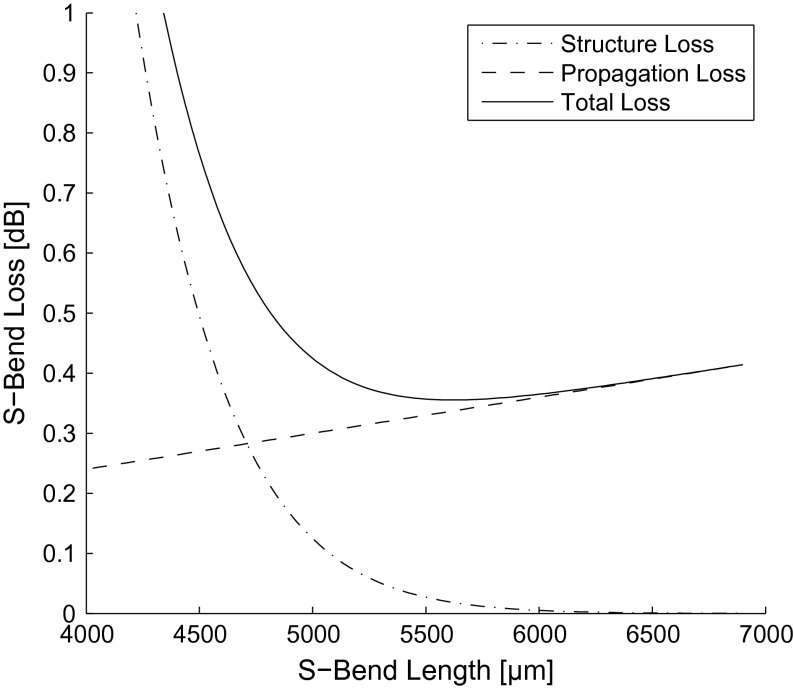
Structure, propagation, and total loss of raised-sine S-bends as a function of S-bend length (*W* = 1000 μm).

Both structure loss and propagation loss need to be considered to obtain optimal device parameters for minimum total loss. While structure loss exponentially decreases with longer S-bend lengths, propagation loss linearly increases with longer S-Bend lengths, as shown in Figure 14. The summation of these loss factors as a function of S-Bend length demonstrates optimal S-bend dimensions required for minimum loss.

Raised-sine S-bends are preferred as the S-bend design of choice when WGs exhibit low propagation loss (<1 dB/cm). If device footprint requirements overshadow minimal device loss, cosine and radial arc S-bends are preferred for low and high NA WGs, respectively, due to their lower structure loss at shorter S-bend lengths. This is important when using WG material with high absorption loss (>1 dB/cm), where both propagation loss and structure loss need to be balanced for optimal device performance. If higher design efficiency is required, exotic S-bend structures, including WG offsets [19,33] and air trenches [34], can be implemented.

S-bend-based WG splitters require adiabatic tapers to reduce loss and minimize size requirements for efficient splitting. Even with the inclusion of adiabatic tapers, overall optical splitting loss increases as the device size is decreased. Multimode interference devices could also be utilized with a smaller footprint [35], but their wavelength-dependence and environmental sensitivity must be taken into consideration for various S-bend applications.

## 6. Conclusion

SM WG S-bends are important structures for realizing minimum optical loss and maximizing optical interconnect density for many applications in optical-integrated devices, including directional control, pitch adjustment, and optical splitting. S-bend structures are thoroughly analyzed to realize optimal designs for SM WGs of various NA. Raised-sine S-bends exhibit the shortest S-bend length requirements necessary for minimum structure loss (<0.1 dB). For builds with smaller footprint requirements, cosine and radial arc S-bends are the most efficient designs for low NA and high NA WGs, respectively.

Optical elastomers were used to fabricate 6-μm SM WGs through the photolithographic process. Propagation loss for SM straights was measured at 0.59 dB/cm. 3 dB alignment tolerances with SMFs were observed at ±3.5 μm. Optical measurements of fabricated S-bends were correlated to theoretical models to obtain radial and transitional loss coefficients. S-bends showed equivalent loss when a quadratic relationship between S-bend width and length was maintained. SM WG straights and S-bends were tested successfully at 10 GBits/s bit error rate testing. Optical splitters fabricated with 1 dB and 3 dB S-bends exhibited splitting ratios of 49:51 ± 2%. Minimal splitting losses were observed when S-bend splitters were fabricated with adiabatic tapers.
